# Muscle RAS oncogene homolog (*MRAS*) recurrent mutation in Borrmann type IV gastric cancer

**DOI:** 10.1002/cam4.959

**Published:** 2016-11-28

**Authors:** Makiko Yasumoto, Etsuko Sakamoto, Sachiko Ogasawara, Taro Isobe, Junya Kizaki, Akiko Sumi, Hironori Kusano, Jun Akiba, Takuji Torimura, Yoshito Akagi, Hiraku Itadani, Tsutomu Kobayashi, Shinichi Hasako, Masafumi Kumazaki, Shinji Mizuarai, Shinji Oie, Hirohisa Yano

**Affiliations:** ^1^ Department of PathologyKurume University School of MedicineKurumeJapan; ^2^ Division of GastroenterologyDepartment of MedicineKurume University School of Medicine KurumeKurumeJapan; ^3^ Discovery and Preclinical Research DivisionTaiho Pharmaceutical Co., Ltd.TsukubaJapan; ^4^ Department of SurgeryKurume General HospitalKurumeJapan; ^5^ Department of RadiologyKurume University School of MedicineKurumeJapan

**Keywords:** Borrmann type IV, gastric cancer, *IGF1R*, *MRAS*, mutation

## Abstract

The prognosis of patients with Borrmann type IV gastric cancer (Type IV) is extremely poor. Thus, there is an urgent need to elucidate the molecular mechanisms underlying the oncogenesis of Type IV and to identify new therapeutic targets. Although previous studies using whole‐exome and whole‐genome sequencing have elucidated genomic alterations in gastric cancer, none has focused on comprehensive genetic analysis of Type IV. To discover cancer‐relevant genes in Type IV, we performed whole‐exome sequencing and genome‐wide copy number analysis on 13 patients with Type IV. Exome sequencing identified 178 somatic mutations in protein‐coding sequences or at splice sites. Among the mutations, we found a mutation in muscle RAS oncogene homolog (*MRAS*), which is predicted to cause molecular dysfunction. MRAS belongs to the Ras subgroup of small G proteins, which includes the prototypic RAS oncogenes. We analyzed an additional 46 Type IV samples to investigate the frequency of *MRAS* mutation. There were eight nonsynonymous mutations (mutation frequency, 17%), showing that *MRAS* is recurrently mutated in Type IV. Copy number analysis identified six focal amplifications and one homozygous deletion, including insulin‐like growth factor 1 receptor (*IGF1R*) amplification. The samples with *IGF1R* amplification had remarkably higher *IGF1R* mRNA and protein expression levels compared with the other samples. This is the first report of *MRAS* recurrent mutation in human tumor samples. Our results suggest that *MRAS* mutation and *IGF1R* amplification could drive tumorigenesis of Type IV and could be new therapeutic targets.

## Introduction

Gastric cancer is the third leading cause of cancer‐associated death worldwide 1. Its incidence is highest in East Asia, Central and Eastern Europe, and South Africa 2. Borrmann classification divides gastric cancer into five types according to macroscopic appearance 3; Borrmann type IV gastric cancer (Type IV) is macroscopically characterized by a grossly thickened and hard wall tumor without marked ulceration or raised margins. The overall 5‐year survival rate of patients with Type IV is 18–28%, and their prognosis is much poorer than that of patients with other types of gastric cancer 4, 5, 6.

Gastric cancer is also divided into intestinal and diffuse types based on histopathological difference 7. Most Type IV are diffuse‐type and are often clinically regarded as almost equal to scirrhous gastric cancer 8. The microscopic feature of scirrhous gastric cancer is the proliferation of poorly differentiated cancer cells or signet ring cells with abundant fibroblasts.

To date, the development of many drugs targeting genetic abnormalities such as the *BCR*‐*ABL* fusion gene, *HER2* gene amplification, *BRAF* gene mutation, and *EML4*‐*ALK* fusion gene have been successful, and the development of drugs targeting genetic abnormalities is considered an attractive strategy for cancer therapy. In gastric cancer, anti–human epidermal growth factor receptor 2 (HER2) antibody is used for HER2‐positive gastric cancer. However, there is only 24% HER2 positivity in gastric cancer 9, and there is an urgent need to identify molecular targets for the remaining majority of gastric cancer cases. Recent whole‐exome and whole‐genome sequencing of gastric cancer and diffuse‐type gastric cancer revealed significant mutation of *TP53*,* PTEN*,* CTNNB1*,* ARID1A*,* PIK3CA*,* FAT4*,* MUC6*,* CTNNA2*, and other genes are in gastric cancer 10, 11, 12, 13, 14, 15. Moreover, *RHOA* is frequently mutated in diffuse‐type gastric cancer 10, 11, 14. Such findings on genetic abnormalities in gastric cancer are expected to facilitate the development of new therapeutic strategies. However, these reports did not contain information on the Borrmann classification, and no report has focused on comprehensive genetic analysis of Type IV to date. Moreover, Type IV accounts for only 7–13% of gastric cancer cases 4, 5, 6, 16, while the frequency of diffuse‐type gastric cancer is about 50% 17, 18, 19, although it varies across geographic regions. Therefore, it is possible that the previously reported whole‐exome and whole‐genome sequencing analyses did not discover characteristic mutations in Type IV, which has distinct histopathological and macroscopic features and confers poorer prognosis than other types of gastric cancer.

In this study, we performed whole‐exome sequencing and genome‐wide copy number analysis of 13 patients with Type IV to elucidate the molecular mechanisms that underlie the oncogenesis of Type IV and to identify new therapeutic targets.

## Materials and Methods

### Patient and sample preparation

This study was approved by the ethics committee of Kurume University and Taiho Pharmaceutical Co., Ltd. Informed consent was obtained from all subjects. Frozen tissue samples of gastric cancer and matched normal tissue, and peripheral blood samples were obtained from 13 patients who had been diagnosed with Type IV and had undergone surgical resection at Kurume University Hospital (Table 1) (discovery set). The superior and inferior portions of the cancer and normal tissues were formalin‐fixed and paraffin‐embedded (FFPE), and FFPE tissue sections were hematoxylin and eosin–stained for evaluation of tumor cell content by a pathologist. The remaining tissues were immediately frozen and stored at −80°C until further processing. Archival FFPE tumor tissues were obtained from 46 patients who had been diagnosed with Type IV and who had undergone surgical resection at Kurume University Hospital (validation set).

**Table 1:** Patient characteristics

Patient	Sex	Age	T stage	N stage	M stage	Histological type	Tumor cell fraction (%)
#1	M	78	T4b	N3a	0	Sig	95
#2	M	60	T4a	N2	0	Por > Muc	40
#3	F	41	T4b	N1	0	Por	60
#4	F	71	T4a	N3a	0	Por > Sig	80
#5	M	58	T4b	N3a	0	Sig	30
#6	M	68	T4b	N2	0	Sig	50
#7	F	77	T4a	N3a	0	Sig	95
#8	F	65	T3	N3a	0	Sig	50
#9	M	81	T3	N1	0	Sig > Por> Tub	90
#10	F	61	T3	0	0	Por > Sig	90
#11	F	53	T4a	0	0	Sig	35
#12	M	65	T3	N1	0	Por > Muc	40
#13	M	68	T4a	N3a	0	Por > Muc	70

M, male; F, female; sig, signet ring cell carcinoma; por, poorly differentiated adenocarcinoma; tub, tubular adenocarcinoma; muc, mucinous adenocarcinoma.

### Exome capture, library construction, and sequencing

DNA fragment libraries were prepared according to the manufacturer's protocol (Fragment Library Preparation, Publication Part Number 4460960; Life Technologies, Tokyo, Japan). Briefly, 3 *μ*g genomic DNA was sheared, end‐repaired, and ligated with primer adaptors. After ligation, PCR amplification (six cycles) was performed to enrich the targeted sequences. The average fragment size of the DNA library was verified by Bioanalyzer (Agilent Technologies, Santa Clara, CA). The fragmented DNA was hybridized using a TargetSeq Exome Enrichment System (Life Technologies). After washing, the captured DNA libraries were amplified using 10 PCR cycles. P1 beads were prepared using a SOLiD EZ Bead Emulsifier (Life Technologies); the emulsion PCR was carried out in a SOLiD EZ Bead Amplifier (Life Technologies) using the E80 setting. The beads carrying the amplified template DNA were purified on a SOLiD EZ Bead Enricher (Life Technologies), and the purified beads were loaded onto a SOLiD 6‐lane FlowChip (Life Technologies) and incubated for 1 hour at 37°C. Then, the beads were sequenced using a 5500xl SOLiD System (Life Technologies).

### Exome data analysis

Short reads were mapped to the human reference genome hg19 using LifeScope software (Life Technologies). The resultant BAM files were processed with IndelRealigner and BaseRecalibrator tools, followed by identification of single‐nucleotide variants (SNV) and small insertions/deletions (indels) by UnifiedGenotyper in GATK‐Lite 20. Single‐nucleotide polymorphisms (SNPs)/indels with frequency ≥ 1% in dbSNP Build 138 or in the Human Genetic Variation Browser database 21 were removed as germline variants. Finally, somatic mutations were filtered according to the following criteria: (1) variant reads on both strands, (2) minimum coverage of 10 and variant reads <1% in the matched blood or adjacent normal tissue if blood was not available, (3) SNV UnifiedGenotyper Mapping Quality ≥40. The identified mutations were annotated using SnpEff 22.

### Microarray‐based copy number analysis

Microarray‐based copy number variation analysis was carried out using CytoScan HD Array (Affymetrix Japan KK, Tokyo, Japan), which consists of >2.4 million copy number markers and approximately 750,000 SNPs. DNA labeling and hybridization were performed according to the manufacturer's instructions. Briefly, 250 ng genomic DNA from tumor, matched normal tissue samples, and blood samples were digested with *Nsp*I for 2 h at 37°C, and then ligated to an adaptor for 12–16 h at 16°C, followed by PCR amplification using a single pair of primers that recognized the adapter sequence, and TITANIUM *Taq* DNA Polymerase (Takara Bio Inc., Shiga, Japan). The PCR products were run on a 2% agarose gel to confirm that the PCR had been performed properly. PCR products were purified using magnetic beads, fragmented using DNase I for 35 min at 37°C, and visualized on a 4% agarose gel to confirm that the fragment sizes ranged from 25 to 125 bp. The fragmented PCR products were subsequently labeled with biotinylated nucleotides using terminal deoxynucleotidyl transferase for 4 h at 37°C. The labeled DNA was hybridized to a pre‐equilibrated CytoScan HD Array for 18 h at 50°C. The arrays were then washed and stained using a GeneChip Fluidics Station 450 and scanned using GeneChip Scanner 3000 7G (Affymetrix). Scanned data files and CEL files were generated using GeneChip Command Console software (Affymetrix) and analyzed using Chromosome Analysis Suite software (Affymetrix) with HapMap reference. The criteria for gene copy number alterations (CNAs) were: (1) copy number state ≥4 for amplifications or 0 for loss in tumor, (2) copy number state = 2 in the matched blood.

### Sanger sequencing

The coding exons of the muscle RAS oncogene homolog *MRAS* (exons 2–6) were amplified according to the Tks Gflex DNA Polymerase protocol (TaKaRa). The reaction mixture contained 10 ng genomic DNA, 1 *μ*mol/L forward and reverse PCR primers (Table S1), and 1.25 U Tks Gflex DNA polymerase. The cycle conditions in the PTC‐225 Thermal Cycler (MJ Research, Waltham, MA) were as follows: 1 min at 94°C for initial denaturation and then 30 cycles at 98°C for 10 sec, 60°C for 15 sec, and 68°C for 1 min, with 7 min at 72°C for postextension. PCR products were separated by agarose gel electrophoresis using Agarose Plate TAE (2%) (Nacalai Tesque, Kyoto, Japan) and gel‐purified using a Wizard SV Gel and PCR Clean‐Up System (Promega, Tokyo, Japan) or E‐Gel SizeSelect Agarose Gel (2%) (Life Technologies). The purified fragments were sequenced using a BigDye Terminator v3.1 Cycle Sequencing Kit (Life Technologies), using the forward or reverse primer for sequencing (Table S1). Subsequently, a purification step was performed using a BigDye XTerminator Purification Kit (Life Technologies). Finally, the PCR products were analyzed in a 3130xl Genetic Analyzer (Life Technologies). Variants detected in both strands were deemed mutations.

### Quantitative real‐time PCR copy number analysis

Quantitative real‐time PCR was performed on an ABI 7900HT System (Life Technologies). PCR for insulin‐like growth factor 1 receptor (*IGF1R*) was carried out using TaqMan Genotyping Master Mix (2×) (Life Technologies), TaqMan Copy Number Assays (20×) (Assay ID; Primer 1, Hs03045229_cn or Primer 2, Hs05365875_cn; Life Technologies), and 20 ng genomic DNA in a final volume of 20 *μ*L. The thermal cycling conditions were 95°C for 10 min, and 40 cycles of 95°C for 15 sec and 60°C for 1 min. The long interspersed nucleotide element‐1 (LINE‐1) repetitive element was used as the endogenous control. PCR for *LINE‐1* was carried out using Power SYBR Green PCR Master Mix (Life Technologies), 400 nmol/L forward primer (5′‐AAAGCCGCTCAACTACATGG‐3′), 400 nmol/L reverse primer (5′‐GCTCCTGAATGACTACTGGG‐3′), and 40 pg genomic DNA in a final volume of 50 *μ*L. The thermal cycling conditions were 95°C for 10 min, and 40 cycles of 95°C for 15 sec and 60°C for 1 min.

The relative copy number of *IGF1R* was calculated using the formula 2 × 2^−ΔΔCt^, where ΔΔC_t_ = (threshold cycle [Ct] of target gene in tumor sample − Ct of LINE‐1 in tumor sample) − (Ct of target gene in reference sample − Ct of LINE‐1 in reference sample). For frozen tumor samples, matched blood DNA was used as the reference sample and was assumed to have a copy number of 2. For FFPE samples, the pooled blood DNA was used as the reference sample.

### mRNA analysis by real‐time PCR

We synthesized cDNA from total RNA using a SuperScript VILO cDNA Synthesis Kit (Life Technologies). Quantitative real‐time PCR was performed on an ABI 7900HT System (Life Technologies). PCR for *MRAS* and *IGF1R* was carried out using TaqMan Gene Expression Master Mix (2×) (Life Technologies, TaqMan Gene Expression Assays (20×) (Assay ID; Hs00171926_m1 and Hs00609566_m1, respectively; Life Technologies), and 10 ng cDNA in a final volume of 50 *μ*L. *ACTB* was used as the endogenous control. *ACTB* PCR was carried out using TaqMan Gene Expression Master Mix (2×) (Life Technologies), 400 nmol/L forward primer (5′‐CCTCGCCTTTGCCGATC‐3′), 400 nmol/L reverse primer (5′‐CGAGCGCGGCGATATCA‐3′), 100 nmol/L fluorescein (FAM) probe (5′‐CCGCCGCCAGCTCACCATG‐3′), and 10 ng cDNA in a final volume of 50 *μ*L. The 2^−ΔΔCt^ method of relative quantification was used.

### Immunohistochemistry

Staining was performed on a Ventana BenchMark XT autostainer using an XT ultraView DAB Kit (Ventana Medical Systems, Tucson, AZ). FFPE sections were stained with primary mouse monoclonal antibodies against IGF1R (1:25; clone 24‐31, GeneTex, Inc., Irvine, CA) for 32 min after 32‐min protease retrieval.

## Results

### Exome sequencing

Using a hybridization capture method for exome enrichment followed by next‐generation sequencing, we sequenced 13 exomes from the tumors and matched blood and/or adjacent normal tissues from the patients with Type IV. Table 1 lists the patient clinicopathological data. Tumor cell fractions in each sample were estimated by microscopic inspection of the top and bottom of each tissue block (Table 1). The average read depth of each exome was 102× (Table S2). On average, 85% of the target sequence was covered by at least 10 reads.

### Somatic mutation

We detected 178 putative somatic mutations, including 162 SNVs and 16 indels, in protein‐coding sequences or at splice sites in the 13 tumors (Table 2).

**Table 2:** Variants and somatic mutations detected in protein‐coding sequences or splice sites in the 13 Type IV samples

	SNVs	Indels
Variants detected in tumor	341,023	9,281
Variants detected in tumor excluding common SNPs	32,353	2,977
Somatic mutations	212	16
Nonsilent or splice site somatic mutations	162	16

Splice site somatic mutations, splice donor or acceptor site mutations in introns within coding sequences.

SNPs, single‐nucleotide polymorphisms; SNV, single‐nucleotide variants.

The 178 mutations included 144 amino acid substitutions, 14 stop mutations, 4 splice site mutations, 9 frame shifts, and 7 in‐frame indels (Table 3, Table S3). These mutations are present in known cancer‐relevant genes: *TP53*,* CDH1*,* ARID1A*, and *RHOA*, which are frequently mutated in gastric cancer 10, 11, 12, 13, 14, 15. Among these, *CDH1* was recurrently mutated in the 13 samples: L214R substitution and a splice site mutation. In addition to the known cancer genes, we found a putative oncogene, *MRAS*, and detected R78W substitution in the tumor from patient #4. *MRAS* R78W is located in an evolutionarily conserved region. We confirmed the *MRAS* R78W mutation by mutation‐specific PCR and Sanger sequencing (Fig. S1, Data S1).

**Table 3:** Number of somatic mutations for each mutation type

Mutaion type	Mutations
Synonymous mutation	50
Amino acid substitution	144
Stop mutation	14
Frame shift	9
Amino acid deletion	3
Splice site mutation^a^	4
Other	4

a Splice site mutation, splice donor or acceptor site mutations in introns within coding sequences.

### Prevalence of MRAS mutation

We also sequenced the *MRAS* coding regions in 46 samples of Type IV by Sanger sequencing. DNA was extracted from macrodissected tumor tissues to remove as much as possible of the nontumor tissue. A total eight nonsynonymous mutations were identified (Table 4). In total, 8 of 46 tumors had *MRAS* mutations, with a frequency of 17% (95% confidence interval, 8–31%). This result indicated that *MRAS* is recurrently mutated in Type IV.

**Table 4:** *MRAS* mutations in 46 Type IV samples (validation set)

Mutation type	*n*	Frequency (%)
A69T	2/36	5.6
E72K	1/31	3.2
L123F	1/27	3.7
V128I	1/29	3.4
R138K	1/27	3.7
S156N and D165N	1/34	2.9
V164I	1/34	2.9

### Expression of MRAS

**Figure 1:**
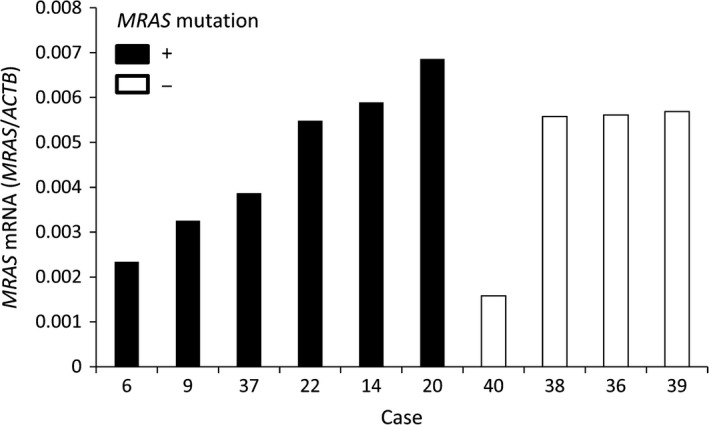
*MRAS* mRNA level in Type IV. Real‐time PCR evaluation of *MRAS* mRNA levels in tumor from samples with or without *MRAS* mutation.

We evaluated mRNA expression of *MRAS* in Type IV by quantitative real‐time PCR. Tumor areas were identified on H&E‐stained sections then macrodissected to remove as much as possible of the nontumor tissue. *MRAS* mRNA was expressed in all the 10 samples. There was no difference in *MRAS* expression between in *MRAS* mutated samples and non mutated samples (Fig. 1).

### Copy number alteration

We also analyzed genome‐wide CNAs in the 13 tumors using CytoScan HD Array. We defined amplification and homozygous deletion as copy number state 4 and 0, respectively, as determined by Chromosome Analysis Suite software (Affymetrix), and detected six focal amplifications and one homozygous deletion. Table S4 shows the genes located in these regions. The 11p13–p11.2 and 15q26.2–q26.3 regions were highly amplified to >10 copies in patient #4. The 15q26 region contains *IGF1R*. Quantitative real‐time PCR validated the *IGF1R* amplification detected by the CytoScan HD Array (Fig. S2).

### Prevalence of IGF1R amplification

**Figure 2:**
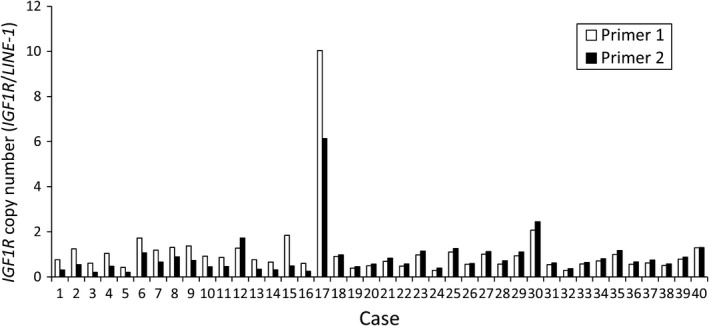
*IGF1R* DNA copy number in the validation set. The DNA copy number of *IGF1R* was evaluated using quantitative real‐time PCR.

**Figure 3:**
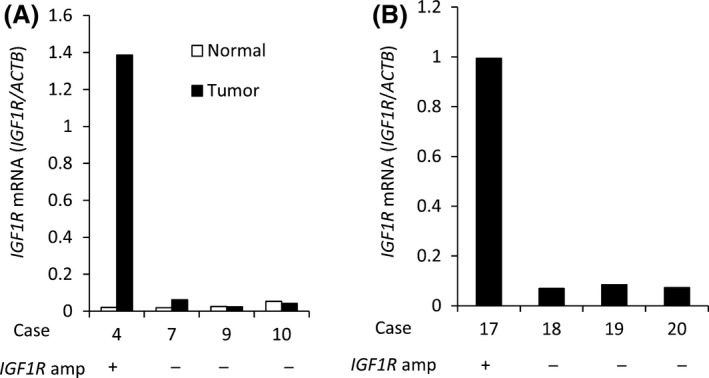
*IGF1R* mRNA level in Type IV. (A) Real‐time PCR evaluation of *IGF1R* mRNA levels in tumor and adjacent normal tissues from samples with or without *IGF1R* amplification (amp) (discovery set). (B) Real‐time PCR evaluation of *IGF1R* mRNA levels in tumor tissues from samples with or without *IGF1R* amplification (amp) (validation set).

**Figure 4:**
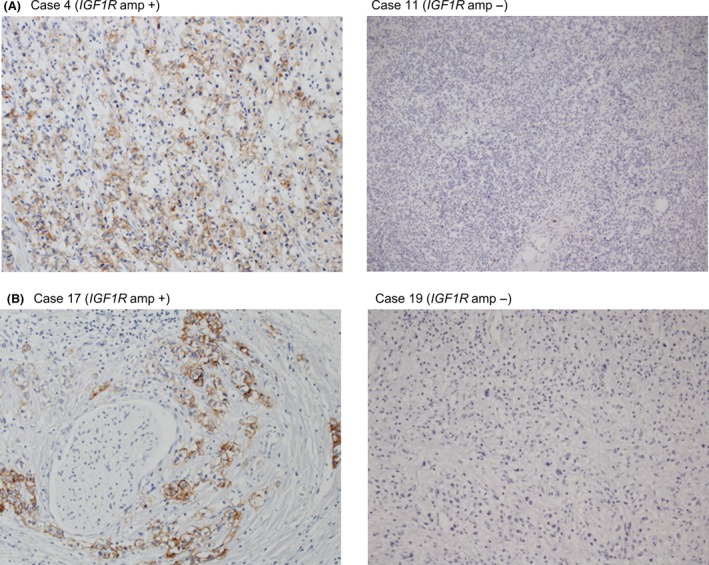
IGF1R protein level in Type IV. (A) IHC evaluation of IGF1R protein levels in tumor tissues from samples with or without *IGF1R* amplification (amp) (discovery set). (B) IHC evaluation of IGF1R protein levels in tumor tissues from samples with or without *IGF1R* amplification (amp) (validation set). IHC, immunohistochemistry.

To estimate the frequency of *IGF1R* amplification in Type IV, we analyzed the copy number in 46 FFPE samples using quantitative real‐time PCR for macrodissected tumor DNA which detected >fourfold amplification in one tumor sample (patient #17; Fig. 2). The tumor sample of patient #4 in the discovery set had both *MRAS* mutation and *IGF1R* amplification, but the tumor sample of patient #17 with *IGF1R* amplification in the validation set did not have MRAS mutation. Copy number data were not obtained for six samples. Combined with the 13 samples analyzed by the CytoScan HD Array, the overall frequency was 3.8% (2/53). We next examined the relationship between *IGF1R* amplification and the expression levels of mRNA and protein. The levels of mRNA expression in the tumor of patient #4 (discovery set) was increased by 69‐fold compared with that in the adjacent normal tissues and by 33‐fold compared with the median in tumors without *IGF1R* amplification (Fig. 3A). Moreover, mRNA expression in the tumor of patient #17 (validation set) was increased by 14‐fold compared with the median of tumors without *IGF1R* amplification (Fig. 3B). The expression level of IGF1R protein in 47 samples (discovery set, patient #4; validation set, 40 samples) was evaluated by immunohistochemistry (IHC). IGF1R protein was expressed in cancer cells in the only two cases with *IGF1R* amplification (Fig. 4); there was no positive staining in the remaining 39 samples (data not shown). These results indicate that *IGF1R* mRNA and protein expression levels in Type IV correlate with *IGF1R* amplification.

## Discussion

Recent whole‐genome and whole‐exome analyses have clarified the genomic characteristics of gastric cancer and have identified new potential therapeutic targets and strategies 10, 11, 12, 13, 14, 15. Type IV accounts for 7–13% of gastric cancer cases and has distinct histopathological and macroscopic features and poorer prognosis than other gastric cancers 4, 5, 6, 16. Consequently, Type IV may have distinctive genetic abnormalities, but no report has focused on the comprehensive genetic analysis of Type IV. To the best of our knowledge, this is the first genomic analysis focusing on Type IV.

We discovered a total of nine nonsynonymous *MRAS* mutations; the mutation frequency in the validation set was 17%, indicating that *MRAS* is recurrently mutated in Type IV. *MRAS* belongs to the Ras family of small GTPases, which includes the prototypic RAS oncogenes *HRAS*,* KRAS*, and *NRAS*. The N‐termini of Ras family members share substantial primary sequence homology, particularly the phosphate‐binding loop (P‐loop) and two switch regions (switch I, switch II) in the G‐domain, which performs the basic function of nucleotide binding and hydrolysis 23. The *MRAS* P‐loop and switch region sequences are identical to that in *HRAS*,* KRAS*, and *NRAS* 23. *HRAS*,* KRAS*, and *NRAS* oncogenic mutations occur most frequently in codons 12, 13, and 61, where the P‐loop and switch II region are located. Codons G12 and Q61 of *HRAS*,* KRAS*, and *NRAS* are equivalent to codons G22 and Q71 of *MRAS*, respectively. Mutation in these *MRAS* codons, that is, G22V, G22K, and Q71L, are active mutations, and transfection of these mutants into cells led to transformation 24, 25, 26, 27, 28, 29, 30, 31, 32. However, these activating mutations have not been found in human tumor samples to date. *MRAS* mutation was reported in melanoma (2 of 121 cases) 33 and lung squamous cell carcinoma (3 of 178 cases) 34, but the frequency was less than 2%. This is the first research to discover *MRAS* recurrent mutation among human tumor samples.

The influence of these mutations on *MRAS* function is unclear. However, *MRAS* A69T is in the switch II region and is equivalent to codon A59T of *HRAS*,* KRAS*, and *NRAS*. *HRAS* A59T results in both decreased GTPase activity and increased nucleotide exchange; transfection of *HRAS* A59T into NIH3T3 cells induced transformation 35. Moreover, recent prospective–retrospective analyses of anti–epidermal growth factor receptor (EGFR) therapy have suggested that the *KRAS* and *NRAS* codon 59 mutations are active 36, 37, 38, 39. These reports suggest that *MRAS* A69T could be an active mutation. On the other hand, *MRAS* codon 69, 72, 78, and 156 mutations occur in an evolutionarily conserved region. Therefore, these mutations could also affect *MRAS* function.

*MRAS* activates the phosphatidylinositol‐3‐kinase (PI3K) and MAPK pathways, extracellular signal–regulated kinase (ERK), c‐Jun N‐terminal kinase (JNK), and p38, and regulates cytoskeletal reorganization 40, 41, cell survival 27, and neuronal cell and osteoblast differentiation. Although little is known about the role of *MRAS* in cancer, it is overexpressed in tumors of the breast, uterus, thyroid, colon, stomach, ovary, lung, kidney and rectum 41. Activated *MRAS* mutants also induce epithelial‐to‐mesenchymal transition (EMT) 31. Recent evidence shows that aberrant activation of EMT plays a crucial role in the tumorigenesis, invasion, and metastasis of various tumors, including gastric cancer 42. In Type IV, cancer cells invade the entire gastric wall, forming an indistinct border 43. Thus, EMT induction by *MRAS* mutation might be one mechanism of the tumorigenesis and invasive ability of Type IV. Functional validation of the *MRAS* mutations we have discovered is warranted.

Previous studies that used whole‐exome sequencing for gastric cancer and diffuse‐type gastric cancer did not report *MRAS* mutation 11, 13, 15; however, The Cancer Genome Atlas (TCGA) did 10. Nevertheless, these reports did not include information on Borrmann classification. Thus, it is possible there were too few Type IV samples for detecting *MRAS* mutation. On the other hand, TCGA reported very few *MRAS* mutations in the 287 gastric cancer patients studied and that the frequency was 0.7% 10. Considering the frequency of *MRAS* mutations in our study was 17%, *MRAS* mutation might be characteristic of Type IV. However, further studies are needed to determine the frequency for other Borrmann types of gastric cancer.

IGF1R, a well‐known cancer drug target, is a transmembrane receptor with tyrosine kinase activity and plays a crucial role in malignant transformation and tumor cell proliferation and survival 44. Previous reports have shown that IGF1R protein was overexpressed 75–77% in gastric cancer tissue 45, 46. TCGA also reported that the frequency of *IGF1R* amplification in the 287 gastric cancer patients was 3.8% 10. However, these reports have not clarified the frequency of IGF1R expression or amplification in Type IV. We found two samples with *IGF1R* amplification and these samples had higher expression levels of mRNA and protein than the other Type IV samples. This indicates that IGF1R could be involved in transformation and tumor cell proliferation in Type IV with *IGF1R* amplification. Therefore, it would be interesting to evaluate IGF1R inhibitors in Type IV with *IGF1R* amplification. In this study, the frequency of *IGF1R* amplification in Type IV was 3.8%, which was similar to that observed in the gastric cancer TCGA study. These results indicate that *IGF1R* amplification is not a gene aberration specific to Type IV.

Previous studies using whole‐exome sequencing in gastric cancer reported an average 50–164 nonsynonymous mutations per case 10, 11, 13, 15. We detected an average of 13 nonsynonymous mutations per case in Type IV; the average number of mutations discovered in this study was smaller than that in previous gastric cancer studies. There are two possible reasons for this difference: Type IV might be a cancer type with few mutations. Using whole‐genome sequencing, Wang et al. reported that, compared with nondiffuse gastric cancer, the genomic characteristics of diffuse‐type gastric tumors were fewer somatic mutations and less chromosome instability 14. Exome sequencing analysis by TCGA also suggested that diffuse‐type gastric tumors had fewer mutations and somatic copy number aberrations as compared with nondiffuse gastric cancer. TCGA proposed a molecular classification dividing gastric cancer into four types, including the genomically stable (GS) tumor type 10. The GS tumor type, which lacks extensive somatic copy number aberrations and has the fewest mutations among the four types, enriches the diffuse type, and diffuse‐type gastric cancer comprises about 75% of the GS tumor type. The report did not contain information on Borrmann classification, but suggested that Type IV was a cancer type with low mutation and somatic copy number aberrations, as most Type IV is a diffuse‐type gastric cancer. Another possibility is the influence of tumor content. As almost all Type IV is characterized by extensive stromal fibrosis, it is difficult to obtain samples with high tumor content. TCGA had also included samples with <60% cancer cells from diffuse gastric cancer in the final data set 10, although they usually analyzed only samples composed of at least 60% cancer cells to yield high‐quality data. Our study included six samples with tumor content <60%, which might have been why we discovered a lower number of mutations than those in previous gastric cancer studies.

We evaluated the frequency of *MRAS* mutation and *IGF1R* amplification using macrodissected tumor DNA to remove as much as possible of the nontumor tissue. However, the possibility that the frequency was underestimated still remains, because low percentage of MRAS mutation alleles or low copy‐number gain of *IGF1R* in the tumor tissues of Type IV with low tumor cell contents could not be detected by Sanger sequencing or qPCR, respectively. Validation by other techniques with higher sensitivity is required to investigate the exact frequency of MRAS mutation and *IGF1R* amplification in Type IV.

In conclusion, this is the first report of the genomic profile of Type IV. In 13 patients with Type IV, we detected a total 178 somatic mutations, which cause amino acid changes or splice site alterations, and six focal amplifications and one homozygous deletion. Among the somatic mutations, it was discovered for the first time that *MRAS* is recurrently mutated, indicating that *MRAS* mutations could drive tumorigenesis of Type IV. Moreover, we detected *IGF1R* gene amplification and that it was correlated with high levels of mRNA and protein expression. Our findings provide insight into the molecular mechanisms underlying the oncogenesis of Type IV and new therapeutic strategies for Type IV.

## Conflict of Interest

E. Sakamoto, H. Itadani, T. Kobayashi, S. Hasako, M. Kumazaki, S. Mizuarai, and S. Oie are employees of Taiho Pharmaceutical Co., Ltd. (Tokyo, Japan). H. Yano received research fund from Taiho pharmaceutical Co., Ltd.

## Supporting information


**Figure S1.** Validation of *MRAS* R78W mutation by mutation‐specific PCR.

**Figure S2.** *IGF1R* DNA copy number in Type IV.

**Table S1.** Primer sequences used in Sanger sequencing.
**Table S2.** Read mapping statistics of exome sequencing.
**Table S3.** Somatic mutations detected in Type IV.
**Table S4.** Gene copy number variations detected in Type IV.

**Data S1.** Method of mutation‐specific PCR for MRAS R78W mutation.
