# Supplementary figures and images for: Muscle RAS oncogene homolog (MRAS) recurrent mutation in Borrmann type IV gastric cancer
Source: Cancer Med. 2016 Nov 28;6(1):235–44. doi: 10.1002/cam4.959 (PMC5269692; doi:10.1002/cam4.959)

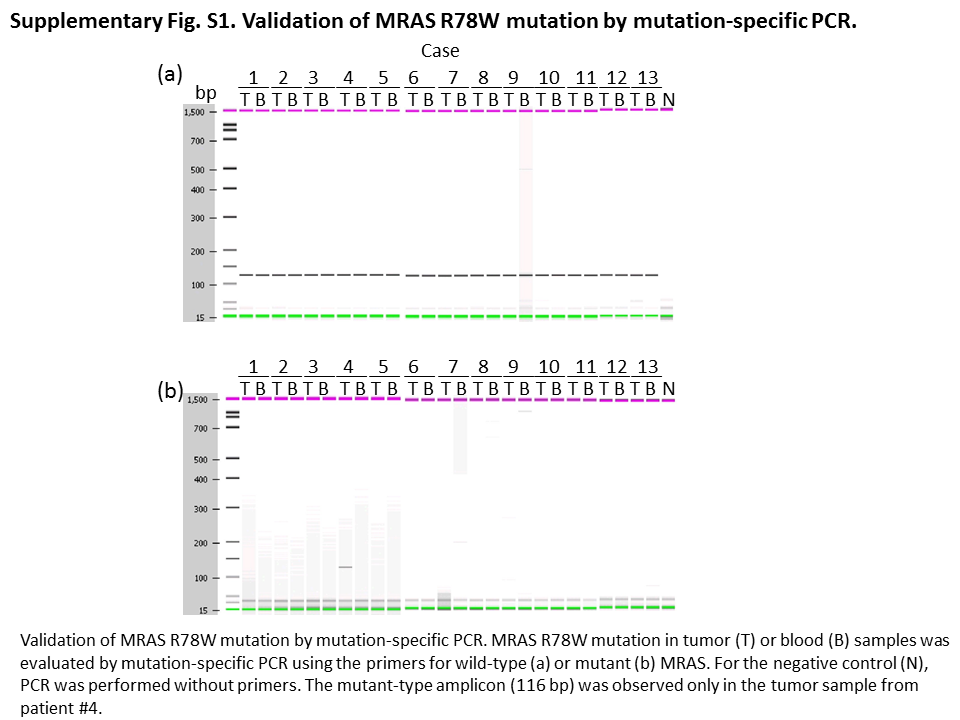

Supplement: Supplementary file 1 — Figure S1. Validation of MRAS R78W mutation by mutation‐specific PCR. [file CAM4-6-235-s001.tif]
