# Supplementary material for: Muscle RAS oncogene homolog (MRAS) recurrent mutation in Borrmann type IV gastric cancer
Source: Cancer Med. 2016 Nov 28;6(1):235–44. doi: 10.1002/cam4.959 (PMC5269692; doi:10.1002/cam4.959)
Supplement: Supplementary file 4 — Data S1. Method of mutation‐specific PCR for MRAS R78W mutation. [file CAM4-6-235-s004.docx]

**Supplementary Doc.S1: Method of Mutation-specific PCR for *MRAS* R78W mutation**

We designed two forward primers with variation in the 3′ nucleotides such that each was specific for the wild-type *MRAS* (5′-GAGGAATTCAGCGCCATGC-3′) or *MRAS* C232T mutation (5′-GAGGAATTCAGCGCCATGT-3′), and one reverse primer (5′-GATAAGCTGGTGGAAGCGGTCCA-3′). PCR was performed with 10 ng genomic DNA, 1 μM forward primer for the wild-type or the C232T mutation, 1 μM reverse primer, and 0.25 U TaKaRa Ex *Taq* (TaKaRa Bio, Shiga, Japan). The amplification products were separated on an Agilent Bioanalyzer using an Agilent DNA 1000 kit (Agilent Technologies, Tokyo, Japan).
